# Supplementary material for: Seroma after Simple Mastectomy in Breast Cancer—The Role of CD4+ T Helper Cells and the Evidence as a Possible Specific Immune Process
Source: Int J Mol Sci. 2022 Apr 27;23(9):4848. doi: 10.3390/ijms23094848 (PMC9101279; doi:10.3390/ijms23094848)
Supplement: Supplementary file 1 [file ijms-23-04848-s001.zip › ijms-1634195-supplementary.pdf]

Characteristics	Study population	Healthy controls
Number (n)	16	15
Age median (range)	73 (48-85)	38 (20-54)
Menopausal status		
premenopausal	1	13
postmenopausal	15	2

Suppl. Table S1: Personal characteristics of the study cohort.

Patient number	Chemotherapy neoadjuvant [yes/no]	Type of axilla surgery	Radiation adjuvant before seroma development [yes/no]
1	yes	axilla dissection	no
2	no	SLNE	no
3 left/right	yes	SLNE left/right	no
4	no	SLNE	no
5	no	no surgery	no
6	no	SLNE	no
7	no	SLNE	no
8 left/right	no	SLNE left/right	no
9	yes	axilla dissection	no
10	yes	axilla dissection	no
11	no	axilla dissection	no
12	yes	SLNE	no
13	no	SLNE	no
14	yes	axilla dissection	no
15	yes	SLNE	no
16	no	axilla dissection	no

Suppl. Table S2: Description of Therapy approaches of patients enrolled in the study.

**Patient number**	**Seroma fine needle aspiration** [x-times]	**1st Aspiration at visit** [visit number]	**2nd Aspiration** [days after 1st aspiration]	**Volume of aspirates** [ml]
1	1x	3	-	75
2	1x	2	-	200
3 left/ right	1x/1x	2	-	185/125
4	15x	2	5	104±52 (20-235)
5	2x	2	17	1st: 20, 2nd:105
6	1x	4	-	75
7	1x	4	-	100
8 left/right	1x	2	-	left: 80, right:160
9	3x	2	14	182±146 (85-350)
10	2x	2	7	1st: 225, 2nd: 80
11	1x	2	-	540
12	2x	2	7	1st: 75, 2nd: 50
13	1x	3	-	40
14	1x	2	-	190
15	1x	2	-	150
16	1x	3	-	23

Suppl. Table S3: Seroma aspirations per patient and visit. Visit numbers: visit 2: 2-3 weeks after surgery, visit 3: 4-6 weeks after surgery, visit 4: 6-12 weeks after surgery, volume of each individual aspiration is shown in ml, for aspiration number >2 the volume is shown as mean ± standard deviation and the minimum as well as maximum volume. Mean aspirate volume: 141.8 ml, Median: 112 ml, SD: 120.6 ml. Chemotherapy preoperative + Seroma postoperative: n=7; out of four got axilla dissection and three SLNE. Chemotherapy preoperative + Seroma ≥100 ml: n=5 out of seven. Chemotherapy preoperative + Seroma ≥100 ml + axilla dissection: n=3 (out of 4) Chemotherapy preoperative + Seroma ≥100 ml + SLNE: n=3 (out of 3) No chemotherapy + Seroma postoperative: n=9; out of six got SLNE, two an axilla dissection and one no axilla surgery. No chemotherapy + Seroma ≥100 ml: n=6 out of nine. No chemotherapy + Seroma ≥100 ml + axilla dissection: n=1 (out of 1) No chemotherapy + Seroma ≥100 ml + SLNE: n=4 (out of 6) No chemotherapy + Seroma ≥100 ml + no axilla surgery: n=1 (out of 1), Seroma + axilla dissection: n=6, Seroma + SLNE: n=9, Seroma + no axilla surgery: n=1, Seroma ≥100 ml: n=11, Seroma ≥100 ml + axilla dissection: n=4 (out of 6), Seroma ≥100 ml + SLNE: n=6 (out of 9), Seroma ≥100 ml + no axilla surgery: n=1,

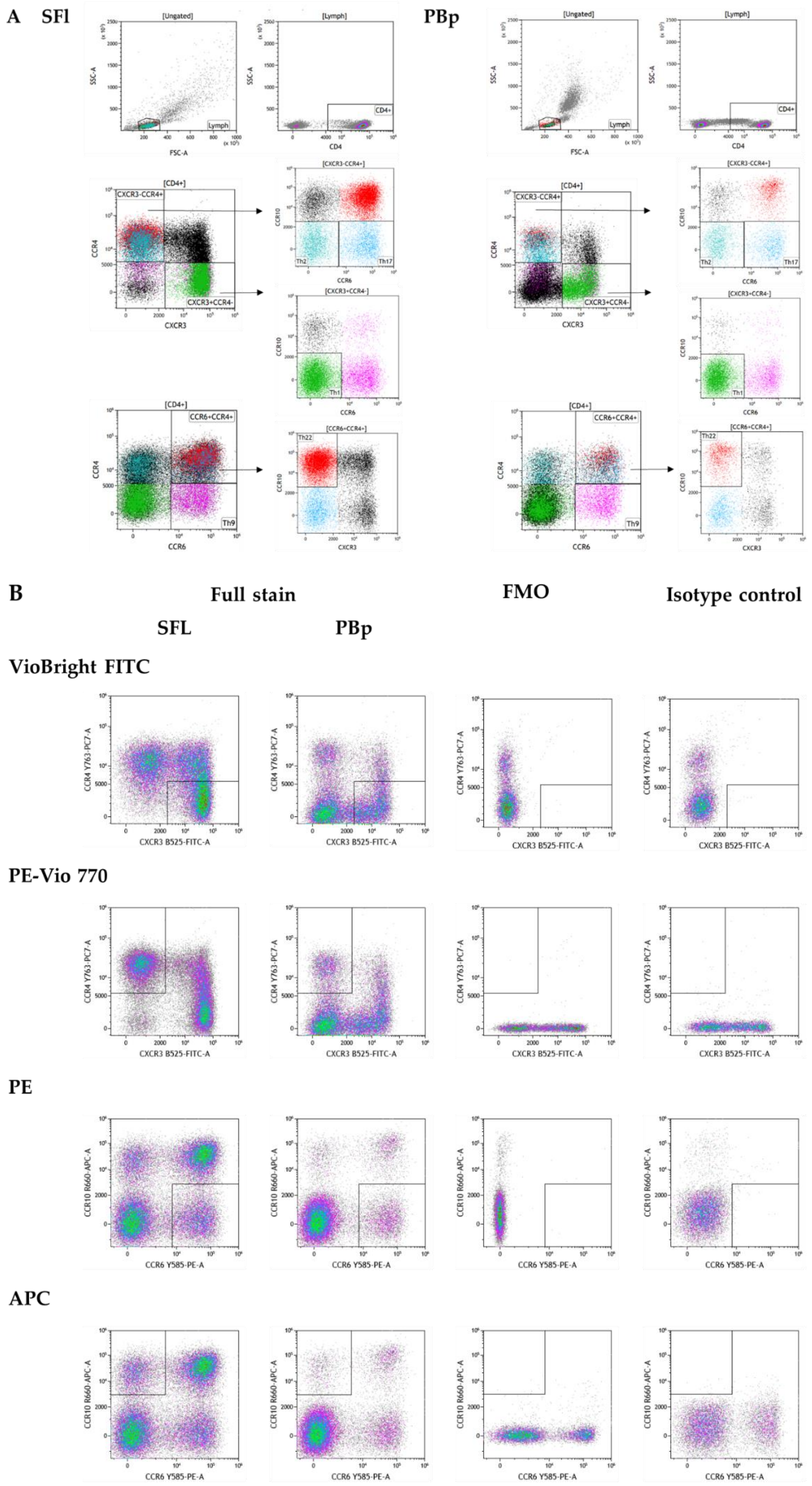

Suppl. Figure S1: Example of flow cytometry analysis of SFl and PBp stained with panel 2. **A)** Gating strategy of panel 2 **B)** FMO and Isotype controls for panel 2.

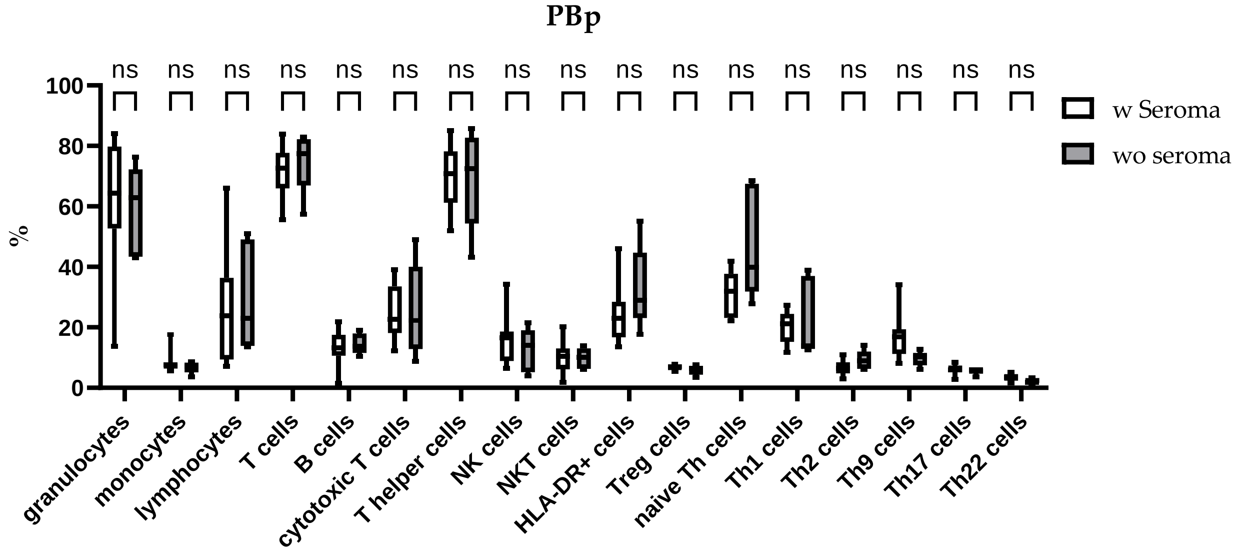

Suppl. Figure S2: Comparison between PBp with (n=12) and without (n=5) seroma formation.
